# IL‐33 delivery induces serous cavity macrophage proliferation independent of interleukin‐4 receptor alpha

**DOI:** 10.1002/eji.201646442

**Published:** 2016-10-11

**Authors:** Lucy H. Jackson‐Jones, Dominik Rückerl, Freya Svedberg, Sheelagh Duncan, Rick M. Maizels, Tara E. Sutherland, Stephen J. Jenkins, Henry J. McSorley, Cécile Bénézech, Andrew S. MacDonald, Judith E. Allen

**Affiliations:** ^1^ School of Biological SciencesUniversity of EdinburghEdinburghEH9 3FLUK; ^2^ Centre for Cardiovascular ScienceUniversity of EdinburghEdinburghEH16 4TJUK; ^3^ Faculty of Biology, Medicine & HealthUniversity of ManchesterManchesterM13 9PTUK; ^4^ Institute of InfectionImmunity & InflammationUniversity of GlasgowGlasgowG12 8TAUK; ^5^ Centre for Inflammation ResearchUniversity of EdinburghEdinburghEH16 4TJUK

**Keywords:** Alternaria, IL‐33, IL‐4, Macrophage, Nematode, Proliferation

## Abstract

IL‐33 plays an important role in the initiation of type‐2 immune responses, as well as the enhancement of type 2 effector functions. Engagement of the IL‐33 receptor on macrophages facilitates polarization to an alternative activation state by amplifying IL‐4 and IL‐13 signaling to IL‐4Rα. IL‐4 and IL‐13 also induce macrophage proliferation but IL‐33 involvement in this process has not been rigorously evaluated. As expected, in vivo delivery of IL‐33 induced IL‐4Rα‐dependent alternative macrophage activation in the serous cavities. IL‐33 delivery also induced macrophages to proliferate but, unexpectedly, this was independent of IL‐4Rα signaling. In a filarial nematode infection model in which IL‐4Rα‐dependent alternative activation and proliferation in the pleural cavity is well described, IL‐33R was essential for alternative activation but not macrophage proliferation. Similarly, during *Alternaria alternata* induced airway inflammation, which provokes strong IL‐33 responses, we observed that both IL‐4Rα and IL‐33R were required for alternative activation, while macrophage proliferation in the pleural cavity was still evident in the absence of either receptor alone. Our data show that IL‐33R and IL‐4Rα promote macrophage proliferation independently of each other, but both are essential for induction of alternative activation.

## Introduction

Over the last 4 years a paradigm shift has occurred in macrophage biology, with the realization that macrophages resident within multiple tissues of the body can self‐maintain without replenishment from blood monocytes 1, 2, 3, 4. In many tissues, stromal derived CSF1 and/or IL‐34 are responsible for the maintenance of resident macrophage numbers during the steady state 5. We found using experimental nematode infections that IL‐4 can promote an increase in tissue macrophage numbers, above homeostatic levels, via proliferation rather than recruitment from the blood 2, 6. The pro‐fibrotic cytokine IL‐13, which is another ligand for IL‐4Rα, can also cause body cavity macrophages to divide in vivo 6. The interaction between IL‐4Rα dependent cytokines and CSF1 is thought to ensure the expansion of the macrophage pool without the influx of inflammatory cells. Indeed, the combinatorial action of both CSF1 and IL‐4 is required to control macrophage numbers within the peritoneal cavity during nematode infection 6.

IL‐33 is a member of the IL‐1 family of cytokines, and is known to promote type‐2 responses 7, in particular through the induction of IL‐5 and IL‐13 secretion from both T cells and innate lymphoid cells (ILCs). IL‐33 has also been shown to indirectly and directly activate eosinophils 8, 9, which are a source of IL‐4 capable of driving the alternative activation of macrophages 10, 11, 12. The ability of IL‐33 to induce type 2 cytokines from multiple cell types makes it unsurprising that IL‐33 has documented roles in the polarization of macrophages to an alternative activation phenotype 13, 14. This is further enhanced by the ability of IL‐33 to upregulate IL‐4Rα expression 13, 14. Although macrophage proliferation has also been described in the context of IL‐33 responses, it is presumed to be dependent upon IL‐33 induction of type 2 cytokines either from ILCs 15 or basophils 16.

In this work we set out to define the contribution of IL‐33 to alternative macrophage activation, and determine whether the impact of IL‐33 extended to macrophage proliferation. We found that IL‐33 delivery in vivo induced both the proliferation and alternative activation of pleural and peritoneal cavity macrophages. While IL‐33‐induced alternative activation was IL‐4Rα‐dependent, IL‐33‐dependent macrophage proliferation occurred in the absence of either eosinophils or the IL‐4Rα. Thus, either IL‐4 or IL‐33 alone was sufficient to drive the proliferation of macrophages in vivo. However, responsiveness to both cytokines was required for macrophage alternative activation during either allergic inflammation or nematode infection.

## Results

### IL‐33 induces macrophage proliferation in vivo

**Figure 1:**
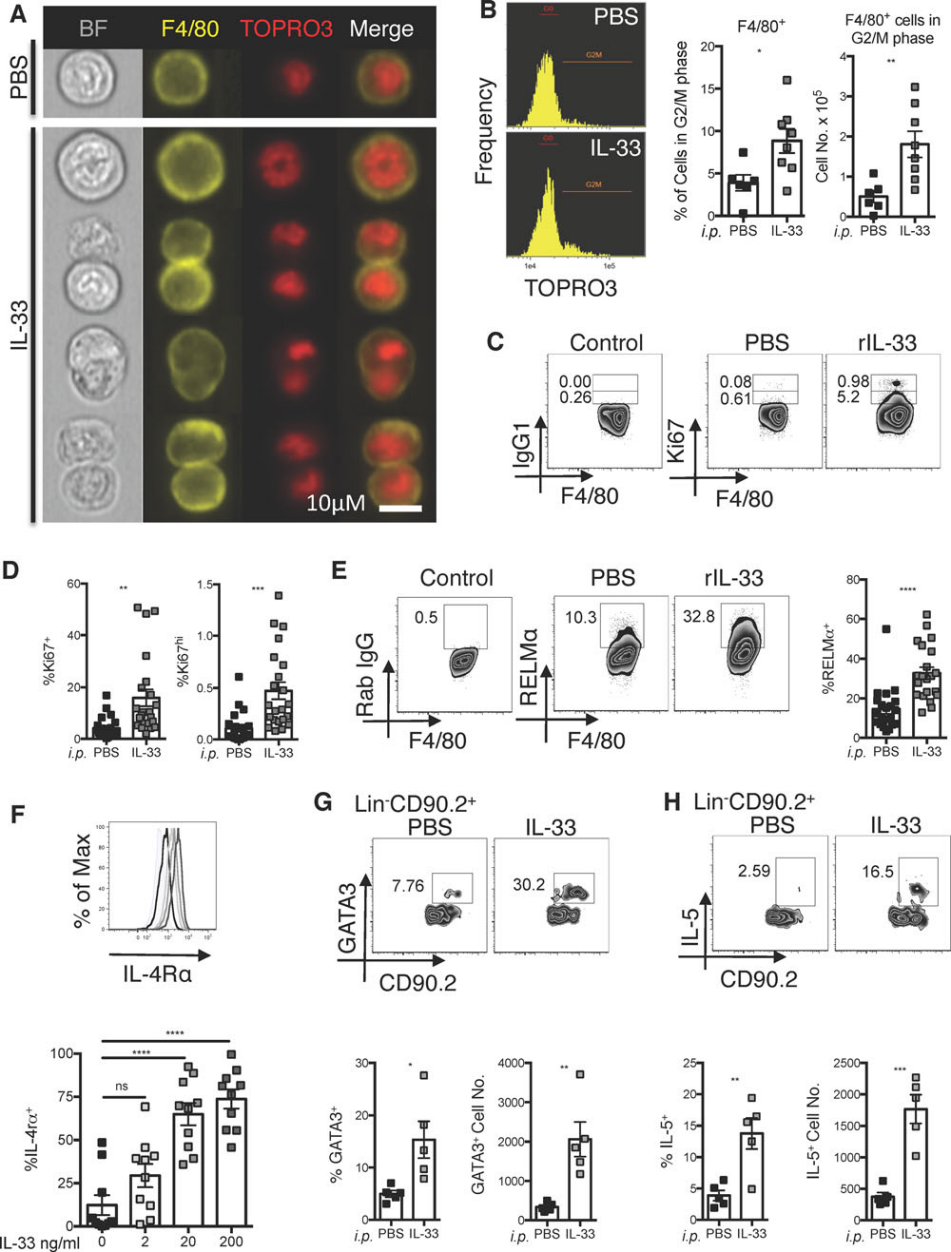
IL‐33 induces body cavity marcrophages to proliferate. ImageStream and flow cytometric analysis of the peritoneal macrophage population harvested following intra‐peritoneal delivery of rIL‐33 or PBS as control. (A) Single cells were discriminated from cell aggregates based on area and aspect ratio. Of those, in focus macrophages were selected based on high gradient RMS of the bright field image and F4/80 expression, intracellular staining with TOPRO3 was used to visualize cellular DNA content, representative ImageStream images of macrophages. (B) Representative histograms of cells in G2/M phase based on TOPRO3 and quantification of cells in G2/M phase shown as percentage of total F4/80^+^ and cell number. (A) Data are representative of two independent experiments with seven mice per experiment; (B) data are combined from two independent experiments with seven mice per experiment, symbols represent individual mice, *n* = 6‐8 per group. (C, D) Flow cytometric analysis showing percent intracellular Ki67 and (E) RELMα within macrophages, isotype control staining (control) is showed for comparison. (C–E) Data are combined from five independent experiments with eight or ten mice per experiment; symbols represent individual mice, *n* = 22 per group. (F) Representative histogram (upper panel) and quantification (lower panel) of IL‐4Rα surface expression on peritoneal cavity macrophages stimulated in vitro with rIL‐33 with indicated concentrations (F) Data are combined from three independent experiments, with three or five mice per experiment, symbols represent culture wells prepared using cells from individual mice, *n* = 10 per group. One way ANOVA with Sidak's multiple comparison test comparing each condition to 0 ng/mL; error bars show mean ±SEM, n.s.: nonsignificant; ****p < 0.0001. (G, H) Intracellular staining for GATA3 and IL‐5 in CD90.2^+^ Lin^−^ (CD11b Ly6G Ly6C Ter119 CD5 B220 CD49b TCRb CD3 NK1.1 CD11c F4/80) peritoneal cavity ILCs harvested following intraperitoneal delivery of rIL‐33. (G and H) Representative density plots (upper panels) and percentage and total number of GATA3 and IL‐5 positive cells (lower panels). (G, H) Data are representative of two independent experiments with ten mice per experiment with *n* = 5 per group. Unless stated otherwise unpaired *t*‐test was used, error bars show mean ±SEM *p < 0.05, **p < 0.01, ***p < 0.001.

To evaluate the capacity of IL‐33 to induce macrophage proliferation, we injected recombinant IL‐33 (rIL‐33) directly into the peritoneal cavity (i.p.) of wild type (WT) BALB/c mice. Proliferation was assessed 48 h later, a time point at which macrophages exhibit robust proliferation following a single delivery of IL‐4. Proliferation was assessed by Imagestream analysis of cellular DNA content and visualized using TOPRO3. A significant increase in F4/80^+^ cells that were in G2/M phase was observed in animals treated with rIL‐33 versus PBS (Fig. 1A and B). Proliferation was also assessed via intracellular staining for the cell cycle protein Ki67 within the F4/80^hi^MHC‐II^lo^ resident macrophage population (Fig. 1C and D). Ki67 staining can reflect cells that have recently proliferated rather than cells that are actively dividing. We thus additionally gated on macrophages which exhibited a high level of Ki67 expression after staining with BD clone B56. Because it has been demonstrated that these cells are in active cell cycle 6, 17, Ki67^high^ staining provided additional confirmation that treatment induced cellular proliferation. Both Imagestream analysis and Ki67 staining demonstrated that IL‐33 was sufficient to induce proliferation of peritoneal macrophages (Fig. 1; see Supporting Information Fig. 1 for macrophage gating strategy). rIL‐33 delivery also resulted in significantly increased expression of RELMα (Fig. 1E), a molecule associated with alternative macrophage activation 18 and known to be one of the most highly IL‐4Rα dependent genes in macrophages 19. We also confirmed in vitro that, as shown previously 13, 14, IL‐33 was able to enhance the expression of IL‐4Rα by F4/80^+^ peritoneal macrophages (Fig. 1F). Furthermore, consistent with published data, two days following injection of rIL‐33 we detected an increase in the percentage expression and number of GATA3^+^ and IL‐5^+^ (Fig. 1G and H) ILC2s within the peritoneal cavity. Delivery of rIL‐33 also increased the numbers of eosinophils within the peritoneal cavity from 2 × 10^5^ to 5 × 10^5^ (*p* < 0.01). These data demonstrated that rIL‐33 delivery can initiate serous cavity macrophage division and alternative activation and suggested that this may occur via induction of type 2 cytokine secreting ILCs and eosinophils acting via enhanced IL‐4Rα signaling.

### IL‐33 promotion of macrophage proliferation does not require eosinophils

**Figure 2:**
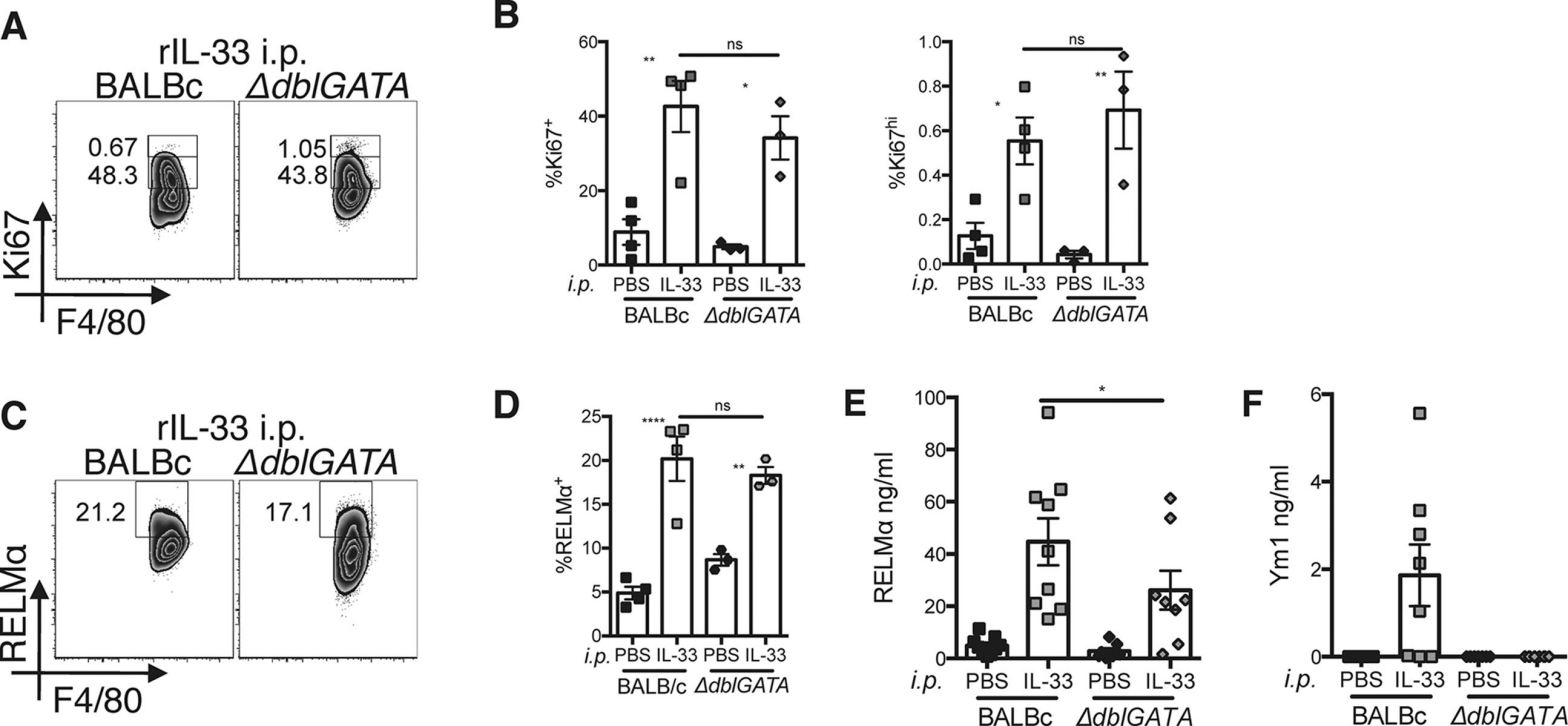
IL‐33 mediated macrophage proliferation does not require eosinophils. Peritoneal cavity macrophages and lavage were harvested following intra‐peritoneal delivery of rIL‐33 into wild type (BALB/c) and eosinophil deficient (*ΔdblGATA)* mice. Flow cytometric analysis of (A) intracellular Ki67 staining, (B) quantification of % Ki67 expression, (C) intracellular RELMα, and (D) quantification of % RELMα expression within macrophage population. (E and F) ELISA of RELMα and Ym1 protein levels in peritoneal lavage. (A–D) Data are representative of two independent experiments with 14 or 19 mice per experiment, symbols represent individual mice *n* = 3–5 per group. One way ANOVA with Sidak's multiple comparison test comparing IL‐33 to PBS treatment for each strain and IL‐33 treatment between strains, error bars show mean ±SEM, n.s.: nonsignificant, *p < 0.05, **p < 0.01 (E, F) Data are combined from two independent experiments with 14 or 19 mice per experiment, *n* = 7–9 per group. One way ANOVA with Sidak's multiple comparison test comparing IL‐33 treatment between strains, error bars show mean ±SEM, *p < 0.05.

The increase in eosinophils and IL‐5 expressing ILCs in the peritoneal cavity following IL‐33 treatment, along with the documented ability of eosinophils to produce IL‐4 10, 11, 12, led us to address whether IL‐33‐dependent macrophage proliferation required eosinophils. We therefore injected rIL‐33 i.p. into eosinophil deficient mice (Δ*dblGATA*) and assessed Ki67 expression within the peritoneal macrophage population. There was no significant difference between BALB/c WT and *ΔdblGATA* mice in the percentage expression of Ki67 at two days following rIL‐33 delivery (Fig. 2A and B). However, there was a minor trend toward a reduction in the percentage of RELMα^+^ macrophages in the *ΔdblGATA* mice (Fig. 2C and D) and a significant reduction in secreted RELMα protein (Fig. 2E). More dramatically, there was a complete failure to induce Ym1 protein within the peritoneal lavage fluid of eosinophil deficient mice treated with IL‐33 (Fig. 2F). These data indicated that IL‐33‐induced proliferation of macrophages did not require eosinophils, but that eosinophils did contribute to alternative macrophage activation. Because RELMα and Ym1 are secreted in response to IL‐4Rα signaling, their reduced protein levels in the *ΔdblGATA* mice suggested that eosinophils are an important source of IL‐4 in mice treated with IL‐33. In contrast, there was no significant change in the amount of macrophage proliferation in the absence of eosinophils, leading us to question our original hypothesis that IL‐33‐mediated proliferation occurs indirectly via downstream activation of the macrophage IL‐4Rα.

### IL‐33 does not require IL‐4Ra to induce macrophage proliferation

**Figure 3:**
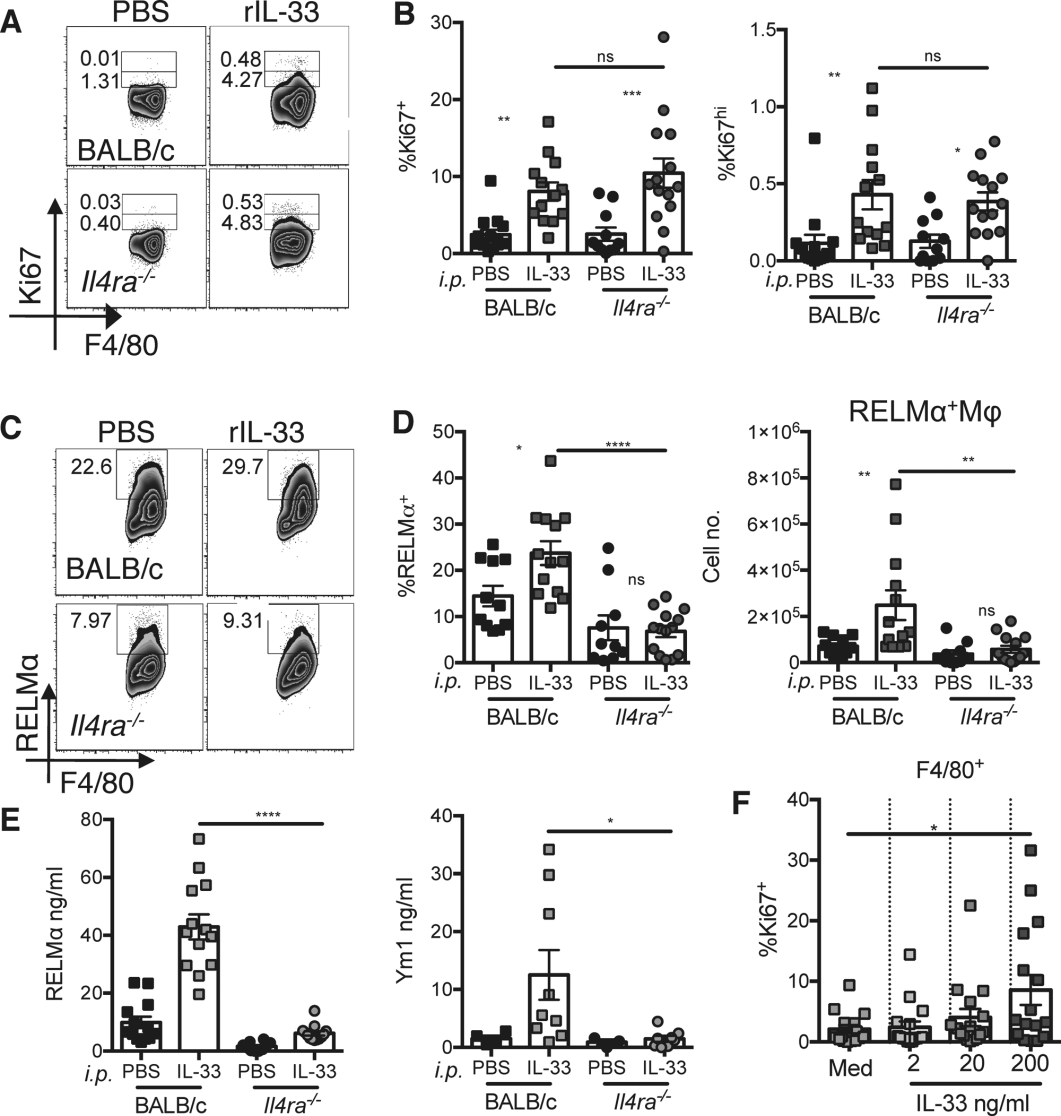
IL‐33 mediated macrophage proliferation does not require IL‐4Rα. Peritoneal cavity macrophages and lavage were harvested following intra‐peritoneal delivery of rIL‐33 into wild type (BALB/c) and IL‐4Rα deficient (*Il4ra^−/−^*) mice. (A–D) Flow cytometric analysis of intracellular (A) Ki67 staining, (B) quantification of % Ki67 expression, (C) of intracellular RELMα, and (D) quantification of % RELMα expression and RELMα^+^ macrophage (Mφ) number. (E) ELISA analysis of RELMα and Ym1 protein levels in peritoneal lavage. (A–E) Data are combined from three independent experiments with 16 or 20 mice per experiment, symbols represent individual mice, *n* = 10–14 per group. One‐way ANOVA with Sidak's multiple comparison test comparing IL‐33 to PBS for each strain and IL‐33 treatment between strains, error bars show mean ±SEM, n.s.: nonsignificant, *p < 0.05, **p < 0.01, *** *p* < 0.001, ****p < 0.0001 (F) Total wild‐type peritoneal cells were cultured in vitro with rIL‐33 with indicated concentrations or medium, % Ki67 expression was determined in F4/80^+^ macrophages by flow cytometry. (F) Data are combined from four independent experiments with three or five mice per experiment, symbols represent culture wells prepared using cells from individual mice, *n* = 11–15 biological replicates per group. One way ANOVA with Sidak's multiple comparison test comparing each dose of IL‐33 to the media control, error bars show mean ±SEM, *p < 0.05.

In order to address whether the ability of IL‐33 to mediate macrophage proliferation required IL‐4 responsiveness, we injected rIL‐33 into the peritoneal cavity of *Il4ra* deficient and WT BALB/c mice, and compared macrophage numbers 48 h later. We found no significant difference between genotypes in the expression of Ki67 within the resident macrophages following rIL‐33 treatment (Fig. 3A and B). In contrast, there was a highly significant reduction in RELMα expression within peritoneal macrophages (Fig. 3C and D) and secreted RELMα and Ym1 protein (Fig. 3E) within the peritoneal lavage fluid of the IL‐4Rα deficient animals, confirming a requirement for IL‐4Rα signaling for induction of these alternative activation markers. Because IL‐33 was still able to promote macrophage proliferation in the absence of IL‐4Rα, we addressed whether IL‐33 could induce macrophage proliferation directly in vitro. We cultured total peritoneal exudate cells (PECs) with increasing concentrations of rIL‐33 and assessed the level of Ki67 within F4/80^+^ macrophages following 2 days of culture (Fig. 3F). A small but significant increase in the percentage proliferation of peritoneal macrophages was seen when cells were cultured with IL‐33 but only at the highest dose of rIL‐33 (200 ng/mL). These data suggested that when provided at a high enough concentration IL‐33 can either act directly on macrophages to induce their proliferation or is able to induce PECs to secrete a downstream macrophage mitogen. We failed to detect an increase in the levels of the known mitogens CSF1, CSF2, and IL‐4 within the culture well (data not shown). The ability of IL‐33 to induce proliferation in vitro, although sub‐optimal, is in contrast to IL‐4, which consistently fails to do so (unpublished observation). Because IL‐4Rα signaling can induce IL‐33R expression 13, 19 we considered the possibility that IL‐33R may act downstream of IL‐4‐driven macrophage proliferation in vivo. To test this possibility, we injected long acting IL‐4 complex (IL‐4c) 20 i.p. into WT BALB/c and IL‐33R deficient animals and assessed proliferation and alternative activation of peritoneal macrophages 48 h later. IL‐4c increased macrophage numbers, Ki67 expression and RELMα expression equally in both strains (Supporting Information Fig. 2). Thus, IL‐4 acts independently of IL‐33R signaling.

### IL‐33 induces pleural cavity macrophage proliferation and alternative activation

**Figure 4:**
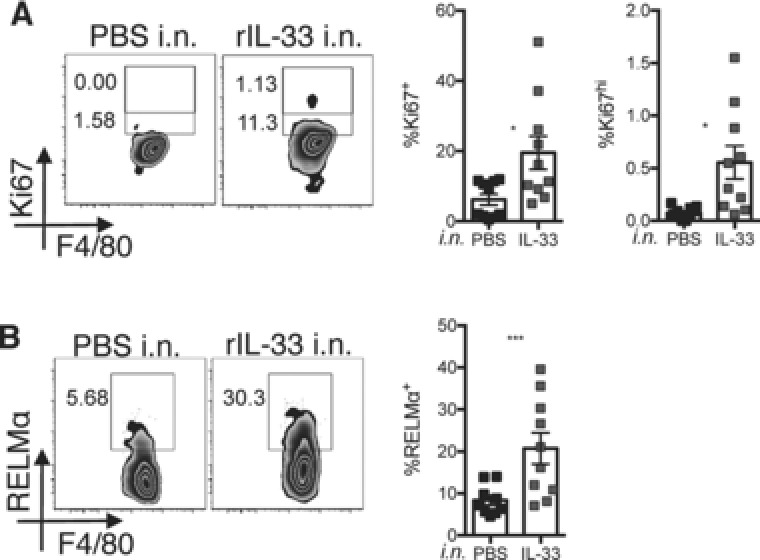
IL‐33 delivered intra‐nasally causes pleural cavity macrophages to proliferate and alternatively activate. Flow cytometric analysis of pleural macrophage population harvested following intra‐nasal delivery of rIL‐33, showing percent intracellular (A) Ki67 and (B) RELMα. Representative density plots (left panels) are from a single experiment representative of two independent experiments with ten mice per experiment. (A, B) Quantitative data are combined from these independent experiments; symbols represent individual mice, *n* = 10 per group. Unpaired *t*‐test, error bars show mean ±SEM *p < 0.05, ***p < 0.001.

We wished to assess whether our observations in the peritoneal cavity applied more broadly to the other serous cavities. We have previously found that allergen delivery induces a profound immune response in the pleural space 21. We thus delivered rIL‐33 by the intra‐nasal (i.n.) route (Fig. 4A and B) and assessed proliferation by Ki67 staining within the F4/80^hi^MHC‐II^lo^ macrophage population of the pleural space. IL‐33 delivered intra‐nasally resulted in an increase in the percentage expression of Ki67 and RELMα within macrophages of the pleural cavity (Fig. 4A and B). These data confirmed that lung derived IL‐33 can influence immune cells within the cavity in which the lungs are contained, and that IL‐33 is able to induce macrophage proliferation at tissue sites other than the peritoneal cavity.

### Alternaria induces macrophage proliferation independently of the IL‐4Rα

**Figure 5:**
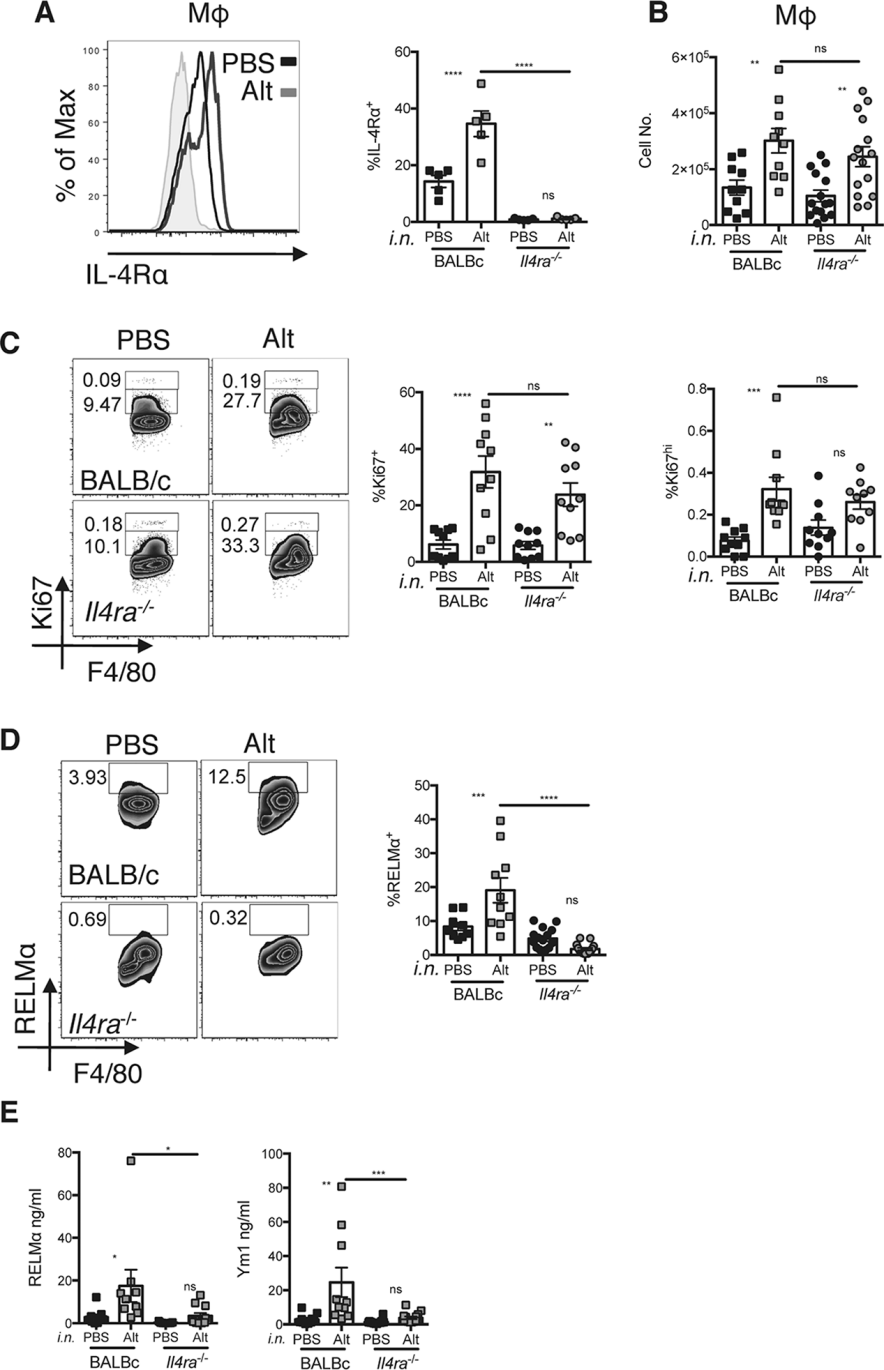
Alternaria can induce pleural cavity macrophages to proliferate but not alternatively activate independently of IL‐4Rα. BALBc and *Il4ra^−/‐^* mice had *Alternaria* extract delivered i.n. and pleural cavity cells were harvested. (A) Pleural cavity macrophage IL‐4Rα expression, (B) Total pleural cavity macrophage number, (C) % Ki67 expression and (D) % RELMα expression within the macrophage population were determined by flow cytometry. (E) RELMα and Ym1 protein levels within the pleural lavage were determined by ELISA. (A) Data are representative of two independent experiments, with 20 mice per experiment, symbols represent individual mice, *n* = 5 per group. (B–E) Data are combined from two to three independent experiments with 10 or 20 mice per experiment, symbols represent individual mice *n* = 10–15 per group. One way ANOVA with Sidak's multiple comparison test comparing Alt to PBS for each strain and Alt treatment between strains, error bars show mean ±SEM, n.s.: nonsignificant, *p < 0.05, **p < 0.01, ***p < 0.001, ****p < 0.0001.

To assess the role of IL‐33 in macrophage proliferation and alternative activation in more complex and relevant settings, we chose two models in which IL‐33 is known to be functional and involve activation of serous cavity macrophages 22, 23, 24. The first model we chose was delivery of an extract of *Alternaria alternata*, a fungal allergen, into the lung. IL‐33 is a major component of pleural immune response that follows pulmonary instillation of *Alternaria sp*. 21. Lung instillation of *Alternaria* extract resulted in a significant increase in the frequency of macrophages expressing the IL‐4Rα within the pleural cavity of BALB/c animals (Fig. 5A), consistent with the ability of IL‐33 to induce IL‐4Rα expression (Fig. 1E and 13). We therefore assessed the contribution of the IL‐4Rα during *Alternaria* induced airway inflammation by comparing the pleural macrophage phenotype at 48 h in WT and IL‐4Rα‐deficient mice. In both genotypes there was a significant increase in the total number of macrophages within the pleural cavity at 48 h following *Alternaria* delivery (Fig. 5B). Furthermore, no significant difference in expression level of Ki67 between *Alternaria* exposed WT and IL‐4Rα‐deficient mice could be detected (Fig. 5C). These data demonstrated that macrophages present within the pleural cavity divide in response to intra‐nasal challenge with a fungal allergen and that this proliferative response occurs independently of IL‐4Rα signaling. We also determined the activation state of the pleural macrophages in these experiments by assessing the intracellular production (Fig. 5D) and global secretion of RELMα and Ym1 proteins into the pleural lavage (Fig. 5E). *Alternaria* instillation significantly increased the levels of both intracellular macrophage RELMα and secreted RELMα and Ym1 within WT mice, but this was significantly reduced in IL‐4Rα‐deficient mice (Fig. 5D and E). Thus, alternative activation, but not proliferation, was reliant on IL‐4Rα in this allergen model. Because these findings mirrored the delivery of IL‐33 into the peritoneal cavity (Fig. 3A–D), we hypothesized that the high levels of IL‐33 induced following *Alternaria* instillation were responsible for these observations.

### IL‐33R signaling is required for the Alternaria‐induced alternative activation of macrophages

**Figure 6:**
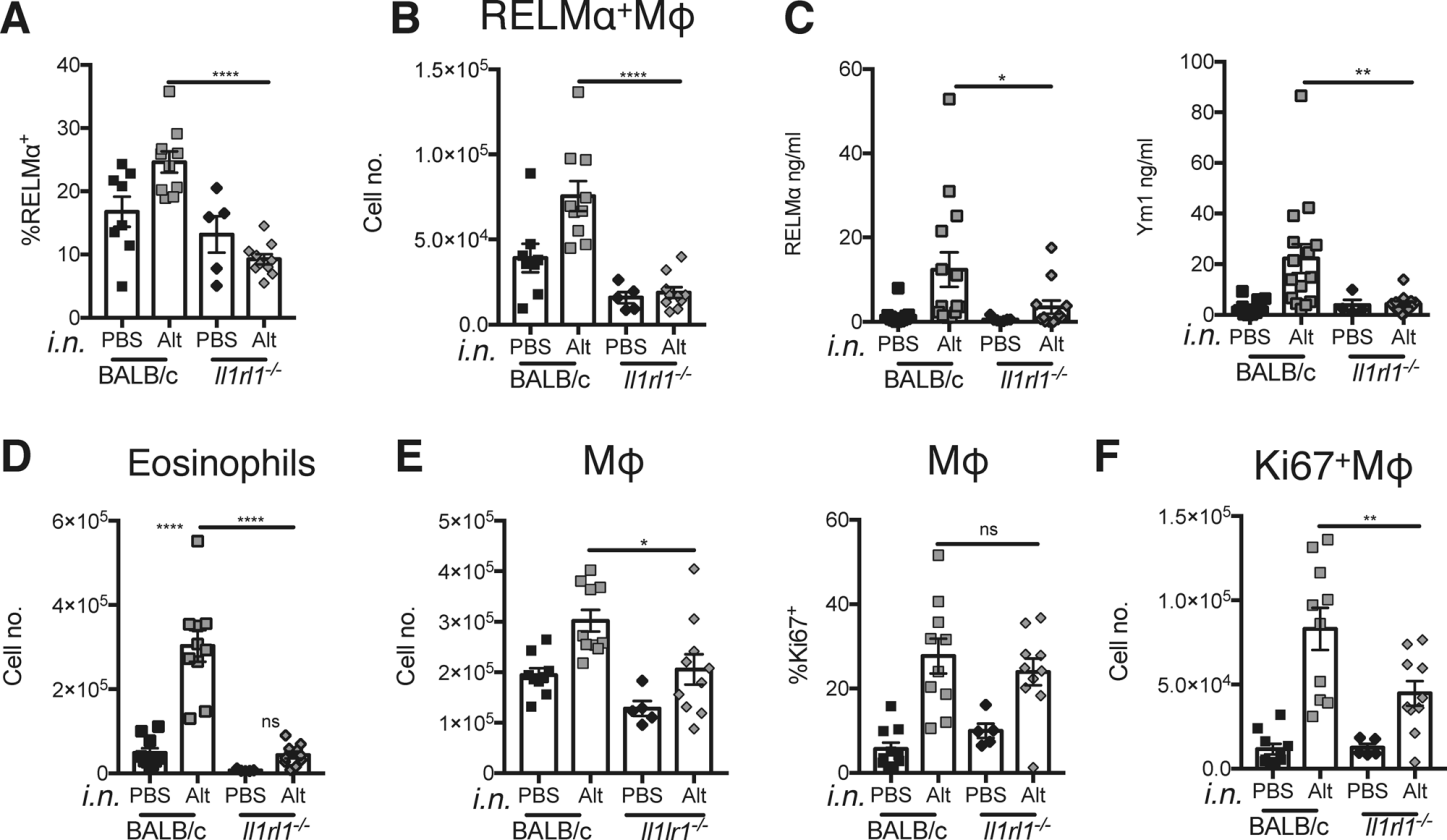
*Alternaria* induces IL‐33R‐dependent alternative activation and IL‐33R‐independent macrophage proliferation in the pleural cavity. BALB/c and *Il1rl1^−/‐^* mice had *Alternaria* extract delivered i.n. and pleural cavity cells were harvested. (A) % RELMα expression and (B) RELMα^+^ macrophage number were determined by flow cytometry. (C) RELMα and Ym1 protein levels within the pleural lavage were determined by ELISA. (D–F) The total number of (D) pleural cavity SSC^hi^SiglecF^+^ Eosinophils, (E) macrophage cell number (left panel) and %Ki67 expression (right panel), and (F) Ki67^+^ macrophage number were determined by flow cytometry. (A–F) Data are combined from two independent experiments with 17 and 18 mice per experiment, symbols represent individual mice *n* = 5–10 per group. One way ANOVA with Sidak's multiple comparison test comparing Alt to PBS for each strain and Alt treatment between strains, error bars show mean ±SEM, n.s.: nonsignificant; *p < 0.05, **p < 0.01, ***p < 0.001, ****p < 0.0001.

To determine if the pleural cavity macrophage response to *Alternaria* was mediated by IL‐33, we delivered the fungal allergen intra‐nasally to both WT and IL‐33R‐deficient mice and assessed macrophage phenotype 48 h later. We found that, in the absence of IL‐33R, there was no induction of the alternative activation marker RELMα by *Alternaria*. Βoth the frequency of expression (Fig. 6A) and total number of RELMα^+^ macrophages (Fig. 6B) were at baseline in the IL‐33R‐deficient cells, resulting in a significant reduction when compared to WT animals (Fig. 6A and B). There was a trend for reduced RELMα protein within the pleural lavage fluid, and significantly reduced Ym1, in the absence of IL‐33R (Fig. 6C), suggesting that an impaired Th2 response had occurred in the absence of IL‐33 signaling, which was further supported by failed recruitment of eosinophils to the pleural cavity (Fig. 6D). Despite the clear defect in type 2 immune responses in the IL‐33R‐deficient mice, there was only a small decline in total macrophage numbers, and no significant difference in the frequency of Ki67^+^ macrophages, between WT and IL‐33R deficient mice at this time point (Fig. 6E). There was, however, a significant reduction in the total number of Ki67^+^ macrophages in the IL‐33R deficient mice (Fig. 6F). These data suggest a deficit in the survival of macrophages in the absence of IL‐33R, which may be an indirect consequence of both reduced IL‐4Rα expression and reduced IL‐4 availability. Thus in the allergy model, IL‐33 receptor signaling is essential for macrophage alternative activation but it's contribution to proliferation is not as direct as that seen with the reductionist IL‐33 delivery model.

### IL‐33R is required for the alternative activation of pleural cavity macrophages during nematode infection

**Figure 7:**
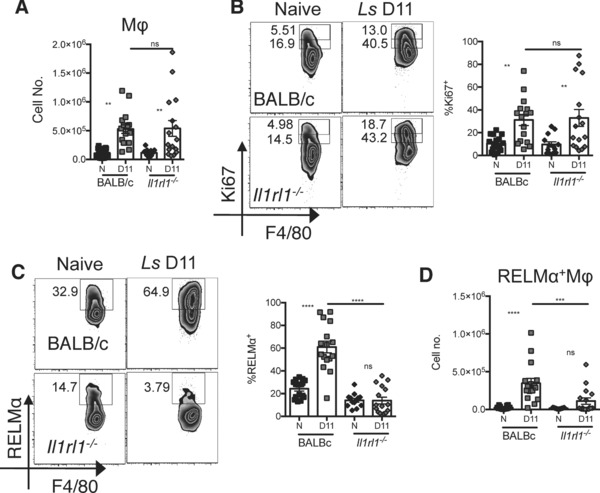
Alternative macrophage activation but not proliferation is IL‐33R dependent during nematode infection of the pleural cavity. BALBc and *Il1rl1^−/‐^* mice were infected subcutaneously with *L. sigmodontis* infective larvae and pleural cavity cells harvested 11 days post infection (D11). Pleural cavity macrophages were analyzed by flow cytometry. (A) Total macrophage number (B) % Ki67 expression, (C) % RELMα expression and (D) RELMα^+^ macrophage number. (A–D) Data are combined from three independent experiments with 17, 21, and 23 mice per experiment, symbols represent individual mice, *n* = 13‐16 per group. One way ANOVA with Sidak's multiple comparison test comparing D11 to Naïve for each strain and D11 infection between strains, error bars show mean ±SEM, n.s.: nonsignificant, *p < 0.05, **p < 0.01, ***p < 0.001, **** *p* < 0.0001.

*Litomosoides sigmodontis* is a nematode parasite that resides in the pleural space and induces a local Th2 response. IL‐4Rα‐dependent proliferation and alternative activation of pleural cavity macrophages are prominent features at day 11 post infection 2, 6. We thus chose this infection model to further address the requirement for IL‐33R in the proliferation or alternative activation of pleural cavity macrophages. We infected WT and IL‐33R deficient mice subcutaneously with *L. sigmodontis* and assessed pleural cavity macrophages 11 days following infection (Fig. 7). There was a significant increase in the number of pleural cavity macrophages in WT animals, but there was no significant reduction in the absence of IL‐33R (Fig. 7A). Consistent with the equivalent increase in macrophage cell number between strains, we found comparable Ki67 expression between the WT and IL‐33R deficient animals 11 days following infection (Fig. 7B). However, while there was a significant increase in the expression of RELMα within the pleural cavity macrophages of WT animals at day 11 following *L. sigmodontis* infection (Fig. 7C), no such increase in either percentage (Fig. 7C) or number (Fig. 7D) of RELMα^+^ macrophages was apparent in IL‐33R deficient animals. Thus, we observed no defect in pleural cavity macrophage proliferation but a failure to induce alternative activation in the absence of IL‐33R in two very distinct model systems, a helminth infection and airway inflammation.

## Discussion

Studies of *Cryptococcus neoformans* infection 25, bleomycin‐induced lung fibrosis 26 and ovalbumin‐induced airway inflammation 13, 27 have demonstrated a role for IL‐33 in the alternative activation of lung derived or alveolar macrophages. These studies suggested that IL‐33 modulates macrophage phenotype by inducing a type 2 cytokine response in accessory cells such as mast cells, basophils, or ILCs. Our studies using administration of recombinant cytokine, a fungal allergen and nematode infection are consistent with these studies and demonstrate that IL‐33‐dependent alternative activation is also reliant on IL‐4Rα in the serous cavities. We further show that eosinophils are a likely source of IL‐33‐induced IL‐4 as they were required for the complete induction of alternative activation within the peritoneal cavity following delivery of exogenous IL‐33. Other cell types are also likely to be IL‐33 targets, in particular ILC2s which produce IL‐5 and IL‐13 in an IL‐33‐dependent manner following *Alternaria* delivery 23. Indeed, IL‐5 produced by ILCs may further enhance the number of IL‐4 producing eosinophils. Our findings taken together with previously published data would indicate that IL‐33 promotes macrophage alternative activation by inducing secretion of IL‐4 or IL‐13 by accessory cells such as eosinophils and ILC2s (Supporting Information Fig. 3). Alternative activation may be further promoted by the ability of IL‐33 to up‐regulate the IL‐4Rα on macrophages.

The interplay between IL‐33R and IL‐4Rα signaling may be particularly important during helminth infection. We observed a lack of IL‐4Rα‐dependent alternative activation during filarial nematode infection of IL‐33R‐deficient mice. IL‐33R deficiency in this model was previously shown to impair splenic clearance of microfilariae from peripheral blood 28, a lifecycle stage that is particularly susceptible to the actions of IL‐4Rα‐activated macrophages 29. As microfilarial clearance is dependent on the spleen 28, it would be of interest to determine which splenic cell type was responsive to IL‐33. In a recent study assessing the role of IL‐33 during experimental malaria, exogenous IL‐33 had a major impact on the spleen 15. Consistent with our findings in the peritoneal cavity, IL‐33 delivery induced alternatively activated macrophages accompanied by expansion of both eosinophils and ILC2s. The authors conclude that ILCs are the major IL‐33 target in the spleen as depletion of the eosinophils did not alter the consequences of exogenous IL‐33 delivery on disease outcome 15.

Far more surprising than the dependent relationship between IL‐4 and IL‐33 was our discovery that each cytokine was able to independently drive macrophage proliferation. In the experimental malaria study described above an increase in macrophage numbers in the spleen was noted following delivery of exogenous IL‐33 but it was not determined whether this was occurring via proliferation or recruitment 15. Similarly, during *Listeria monocytogenes* infection, hepatocyte derived IL‐33 was found to increase macrophage proliferation in the liver 16, and assumed to be due to an amplification loop acting via basophil production of IL‐4. It will be important to address in the context of *Listeria* whether IL‐33 can act independently of IL‐4 to induce macrophage proliferation. Delivery of *Alternaria* intra‐nasally resulted in the proliferation of pleural cavity macrophages in both IL‐4Rα deficient and IL‐33R deficient animals, suggesting that either receptor may be sufficient.

Our data do not allow us to state conclusively that IL‐33 released following *Alternaria* delivery acts directly on the macrophage to cause division. We have previously shown that IL‐4‐driven macrophage proliferation is independent of the requirement for CSF1R signaling and that pleural cavity macrophage proliferation during *L. sigmodontis* involves both CSF1 and IL‐4Rα 6. CSF1 may thus be the mitogen induced by *Alternaria* delivery that results in the proliferation of pleural cavity macrophages, independently of both IL‐4Rα and IL‐33R. Others have described the ability of IL‐33 to induce the proliferation of B1‐cells, a cell population that makes up a large proportion of the immune cells within the serous cavities 30. More recently, it has been shown that sorted B1b cells placed into culture with IL‐33 will secrete CSF2 (GM‐CSF) 31 which leads to the alternative possibility that IL‐33 may induce CSF2 from B‐cells within the serous cavities, which then contribute to the proliferation of resident serous macrophages. Future experiments will be required to determine whether the action of IL‐33 on macrophages requires cell‐intrinsic expression of the IL‐33R and whether there is requirement for B‐cell derived CSF2 in the IL‐33 driven macrophage proliferation system. Unraveling the contribution of CSF2 or CSF1 to IL‐33 driven macrophage proliferation will be critical, as pathology associated with chronic Th2 settings are characterized by elevated IL‐4 and IL‐33 32. If both cytokines are independently driving macrophage proliferation, this could prove to have serious consequences. However, IL‐4 exposure in vivo results in the down‐regulation of *Csf1r* gene expression on macrophages 6 potentially mitigating the combined effect if CSF1 is involved.

Finally, the data we present here demonstrates a close interaction between an inflammatory response occurring in the lung, and the activation of immune cells within the pleural cavity. Our findings, both with IL‐33 delivery and *Alternaria* instillation, demonstrate that airway inflammation triggers a concurrent inflammation within the pleural space, which in turn modulates macrophage phenotype. A close interaction between lung infection/inflammation and pleural cavity activation is likely to prove relevant to a wide range of conditions. For example 4–10% of all pulmonary tuberculosis cases results in pleural effusion 33, 34. The activation of immune cells within the pleural space during this infection is thought to be dependent upon the presence of mycobacterium antigens within the pleural cavity 34, 35, highlighting the ability of lung derived antigens to traffic to the pleural space. The pleural cavity is also a known site at which proximal lung cancers metastasise, and can result in malignant pleural effusion 36. Our new data show that IL‐33 delivery in vivo can induce the proliferation of macrophages independently of IL‐4Rα signaling and serve to highlight an important but often overlooked interaction between the lungs and the serous cavity in which they reside.

## Materials and methods

### Animals, infections, and airway allergy models

Experiments were performed using BALB/c, BALB/c *Il4ra* ^−/−^, BALB/c *ΔdblGATA* 37, and BALB/c *Il1rl1* ^−/‐^ animals that were maintained under specific pathogen–free conditions at the University of Edinburgh or University of Manchester Animal Facilities and used at 6–12 weeks of age. Mice were sensitized intra‐nasally with 50 μg *Alternaria alternata* extract (Greer) in 50 μL dPBS (Sigma), 50 μL dPBS alone at time 0, or 200 ng rIL‐33 (Peprotech) in 50 μL dPBS at time 0 and again at 24 h, all intra‐nasally sensitized animals were culled at 48 h following initial instillation. Mice were infected via sub‐cutaneous injection into the scruff with 30 *Litomosoides sigmodontis* L3s in 200 μL of RPMI 1640 (Invitrogen) containing 5% horse serum, or vehicle alone and all animals were culled 11 days later. Mice were injected intra‐peritoneally with 500 ng rIL‐33 (Peprotech), or 5μg rIL‐4 (Peprotech) complexed to 25 μg anti‐IL‐4 antibody (clone 11B11; BioXcell) in dPBS (Sigma), or dPBS (Sigma) alone, peritoneal cells were harvested after 48 h. All experiments were approved under a Project License granted by the Home Office (UK) and conducted in accordance with local guidelines.

### Cell isolation and culture

Pleural and peritoneal exudate cells were isolated via flushing the cavity with RPMI 1640 (Invitrogen) containing 50 U/mL penicillin, 50 μg/mL streptomycin (Invitrogen) and 2 mM L‐Glutamine (Invitrogen), 1 mL of the first 2 mL of wash was frozen for later ELISA analysis. Erythrocytes were removed by incubating with red blood cell lysis buffer (Sigma). Cellular content of the cavities was assessed by cell counting using a Cellometer ® Auto T4 Cell Counter (Nexcelom Bioscience) in combination with multicolor flow cytometry. For detection of intracellular cytokines in Fig. 1, cells were stimulated for 4 h at 37°C with PMA (phorbol myristate acetate; 0.5 μg/mL; Sigma) and ionomycin (1 μg/mL; Sigma) including brefeldin A (10 μg/mL; Sigma) for the last 3 h of incubation. Cells were then surface stained, fixed and permeabilized as below. In vitro stimulation: Peritoneal exudate cells were isolated from naïve BALB/c or naïve *Il1rl1^−/−^* animals as described above, spun down and seeded as biological replicates in 96 well plates at 1 × 10^6^/mL in 200 μL of RPMI (Invitrogen) containing 10% Fetal Bovine Serum, 50 U/mL penicillin, 50 μg/mL streptomycin (Invitrogen), and 2 mM L‐Glutamine (Invitrogen). 2, 20, or 200 ng/mL rIL‐33 (Peprotech) was included in each well, cells were incubated at 37°C, 5% CO_2_ for 40 h. Supernatants were removed, and cells were stained within the same plate for analysis by flow cytometry.

### Flow cytometry

Samples were washed twice in PBS prior to staining with LIVE/DEAD (Life Technologies) for 10 min at room temperature. Samples were then blocked with 5 μg/mL anti CD16/32 (clone 2.4G2; produced in house) and heat‐inactivated mouse serum (1:10) in FACs Buffer (dPBS containing 0.5% BSA (Sigma), 2mM EDTA (Sigma)) cell surface markers for several different cell populations were detected by addition of various flourochrome‐conjugated antibodies (See Supporting Information Table I for list of antibodies used) for 20–30 min on ice. Samples were washed in FACs buffer prior to intracellular staining which was performed following fixation in either 2% formaldehyde for 10 min at room temperature or 1× Fixation/Permeabilization buffer (eBioscience) overnight followed by permeabilization using 1× permeabilization buffer (eBioscience). Antibodies to detect intracellular proteins (including Ki67 clone B56 from BD) were incubated with samples on ice in permeabilization buffer (eBioscience) for 30 min. Samples were acquired using a BD LSR II. Macrophages were gated as CD19^−^Ly6G^−^SigF^−^TCRβ^−^F4/80^hi^MHC‐II^lo^, Eosinophils were gated as SigF^+^SSC^hi^MHC‐II^−^, ILC were gated as CD3^−^CD11c^−^B220^−^Ly6G^−^Ly6C^−^F4/80^−^CD11c^−^NK1.1^−^Ter119^−^CD5^−^CD11b^−^CD49b^−^CD45.2^+^CD90.2^+^.

### ImageStream

Samples were blocked with 5 μg/mL anti CD16/32 (clone 2.4G2; produced in house) in ImageStream buffer (dPBS containing 1% FCS (Sigma), 2mM EDTA (Sigma)). Cells were stained for F4/80 PE (Clone: BM8 (BioLegend)) for 25 min at 4°C. Samples were washed in ImageStream buffer and fixed in 1% paraformaldehyde for 10 min at room temperature followed by permeabilization with Fixation/ Permeabilisation buffer (eBioscience) for 30 min at 4ºC and washed with 1× permeabilization buffer (eBioscience). Samples were stained with TOPRO3 (1:2000) for 10 min at room temperature and washed once with FACS buffer ready to acquire on the ImageStream. Data acquisition was performed on ImageStream^X^ (Amnis/EMD Millipore, Seattle, WA) equipped with 405, 488, 561, and 642 nm lasers. Single cells were discriminated from cell aggregates based on area and aspect ratio, in focus macrophages were selected based on high gradient RMS of the bright field image and F4/80 expression. The F4/80+ gate was drawn based on an FMO control and a minimum of 5000 F4/80+ cells were collected run at a low flow rate setting. Images of cells were acquired with a ×40 objective including bright field images (Channels 1 & 9; 430–480nm) F4/80 PE (Channel 3; 560–595nm), TOPRO3 (Channel 11; 660–740nm). All data analysis was performed using the IDEAS® software version 6.

### Antigen preparation

*Alternaria* extract (Greer) (Alt) was dissolved in PBS and filter sterilized.

### ELISAs

Sandwich ELISAs were used to detect RELMα (Peprotech) and Ym1 (R&D Systems) within the peritoneal lavage and pleural lavage fluid. Plates were developed using 2‐part TMB reagent (KPL & Biolegend).

**Statistical analysis** was performed using Prism 6 for Mac OS X (v6.0d, GraphPad Software). Statistical tests used are described in each figure legend.

## Conflict of interest

A.S.M. and F.S. are members of MCCIR, which is a joint venture between the University of Manchester, AstraZeneca, and G.S.K. All other authors declare no financial or commercial conflict of interest.

**Abbreviations**
ILCs: innate lymphoid cells
i.p.: intra‐peritoneal
i.n.: intra‐nasal
PECs: peritoneal exudate cells

## Supporting information

As a service to our authors and readers, this journal provides supporting information supplied by the authors. Such materials are peer reviewed and may be re‐organized for online delivery, but are not copy‐edited or typeset. Technical support issues arising from supporting information (other than missing files) should be addressed to the authors.

Supplementary Figures 1–3 and Table 1

Peer review correspondence
