# Supplementary material for: IL‐33 delivery induces serous cavity macrophage proliferation independent of interleukin‐4 receptor alpha
Source: Eur J Immunol. 2016 Oct 11;46(10):2311–21. doi: 10.1002/eji.201646442 (PMC5082546; doi:10.1002/eji.201646442)
Supplement: Supplementary file 1 — Supplementary Figures 1–3 and Table 1 [file EJI-46-2311-s001.pdf]

European Journal of Immunology

Supporting Information
for
DOI 10.1002/eji.201646442

Lucy H. Jackson-Jones, Dominik Rückerl, Freya Svedberg, Sheelagh Duncan, Rick M. Maizels, Tara E. Sutherland, Stephen J. Jenkins, Henry J. McSorley, Cécile Bénézech, Andrew S. MacDonald and Judith E. Allen

IL-33 delivery induces serous cavity macrophage proliferation independent of interleukin-4 receptor alpha

© 2016 The Authors. *European Journal of Immunology* published by WILEY-VCH Verlag GmbH & Co. KGaA, Weinheim.

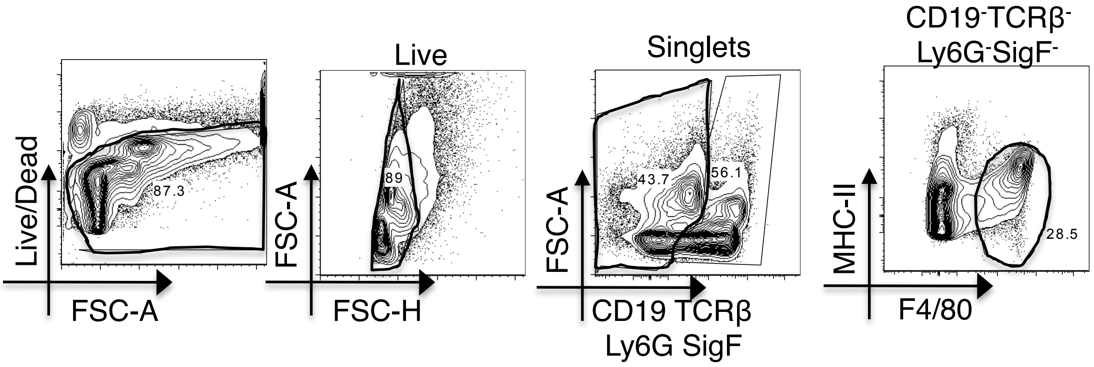

Supplementary Figure 1. Gating strategy used to define macrophages in the serous cavities

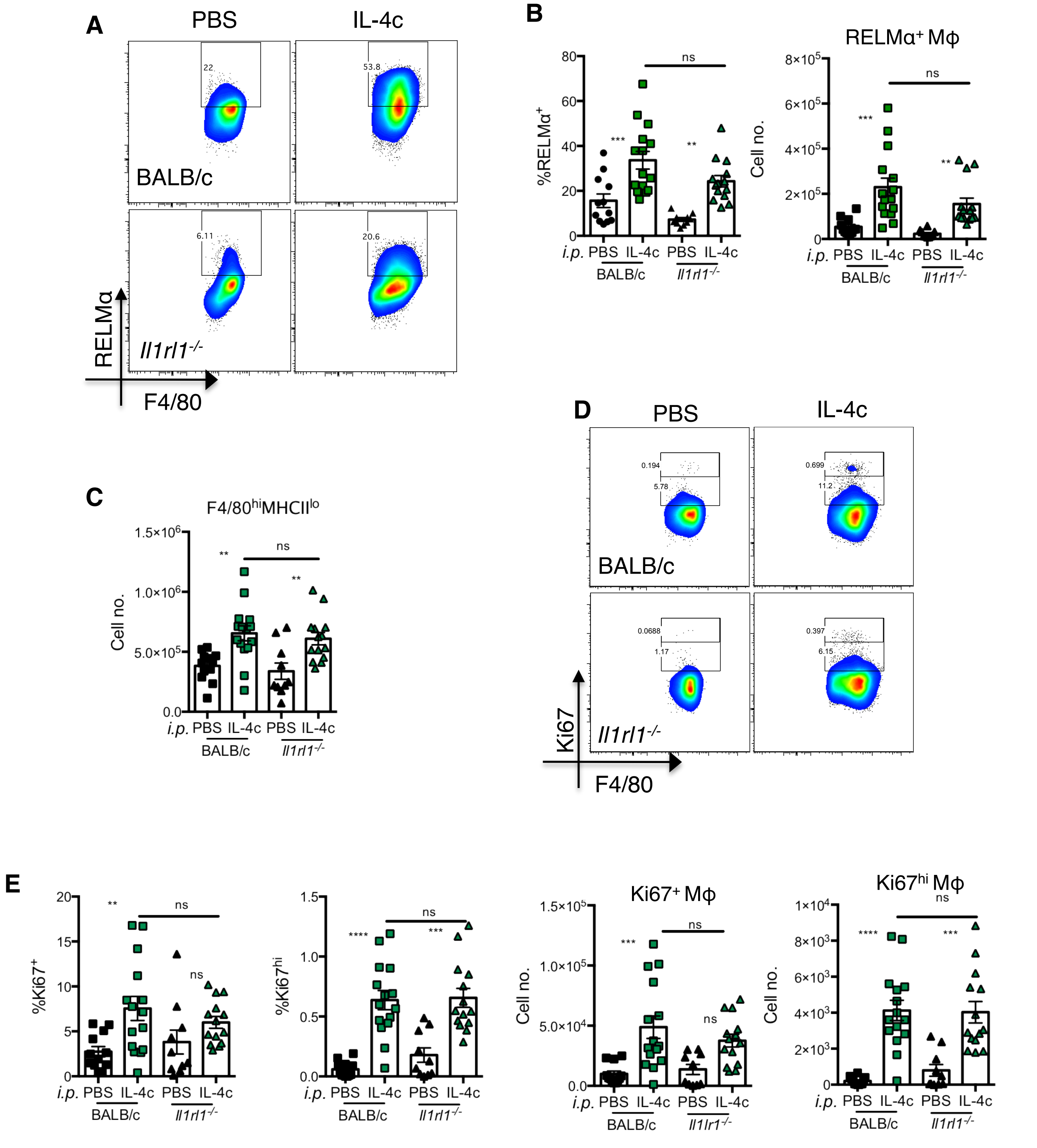

Supplementary Figure 2. Exogenous delivery of IL-4c can induce serous cavity macrophages to alternatively activate and proliferate independently of IL-33R. 5μg of IL-4c was delivered i.p. into BALB/c and *Il1rl1*$^{-/-}$ mice, peritoneal macrophages were harvested 48h later. (A and B) %RELMα expression and RELMα^{+} macrophage number were determined by flow cytometry. (C) Total macrophage cell number were determiend by flow cytometry. (D and E) %Ki67 expression and number of Ki67^{+} and Ki67^{hi} macrophage as determined by flow cytometry. Data are pooled from 3 independent experiments, symbols represent individual mice, n=10-15 per group. One way ANOVA with Sidak's multiple comparison test comparing IL-4c to PBS for each strain and IL-4c treatment between strains, error bars show mean ±SEM, n.s.= non significant, *= P<0.05, **=P<0.01, ***=P<0.001, ****=P< 0.0001

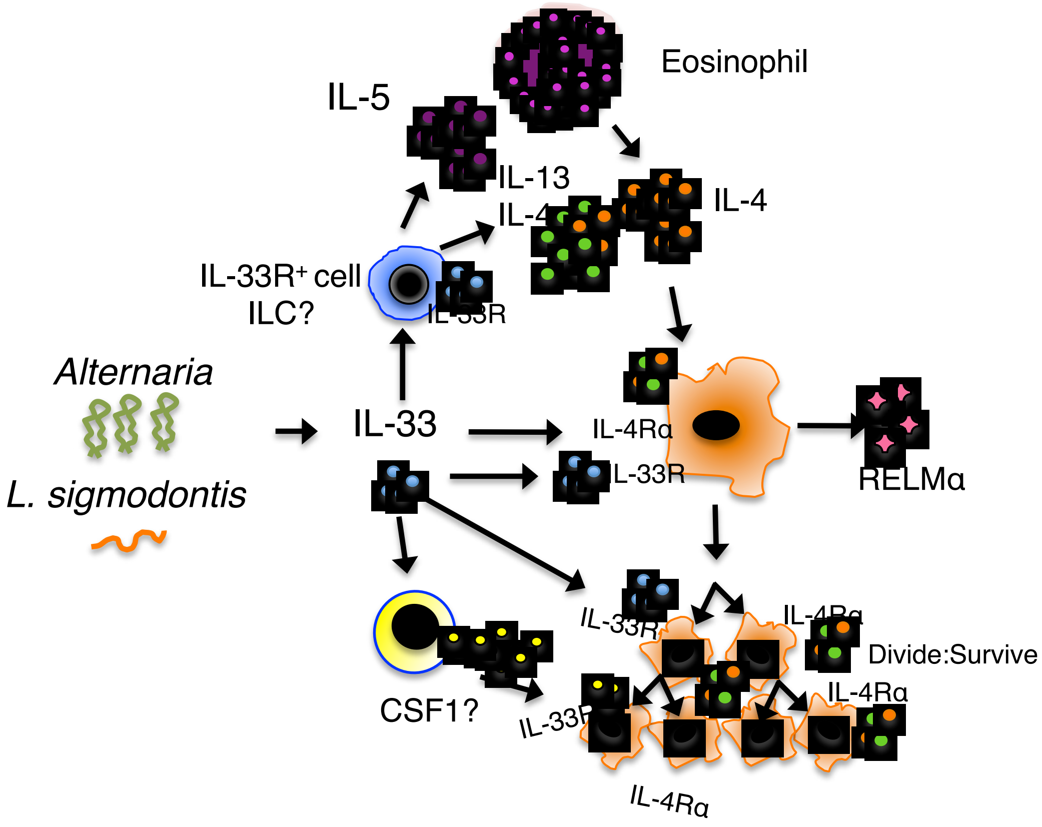

Supplementary Figure 3. Graphical summary of data presented

Antigen	Conjugate	Supplier	Cat Number	Isotype	Clone
**Ly6C**	BV570	BioLegend	128029	Rat IgG2c k	HK1.4
**CD11b**	BV711	BioLegend	101241	Rat IgG2b, k	M1/70
**CD11c**	BV605	BioLegend	117334	Armenian Hamster IgG	N418
**I-A/I-E**	AF780	eBiosciences	47-5321-82	Rat IgG2b k	M5/114.15.2
**F4/80**	PECy7	eBiosciences	25-4801-82	Rat IgG2a, k	BM8
**CD19**	BV421	Biolegend	115538	Rat IgG2a k	6D5
**SigF**	BV421	BD Pharm	562681	rat IgG2a	E50-2440
**Ly6G**	BV421	BioLegend	127627	Rat IgG2a k	1A8
**TCR beta**	Pacific Blue	Biolegend	109226	Armenian Hamster IgG	H57-597
**Ki67**	FITC	BD Pharm	556026	Ms IgG1 k	B56
**RELMa**	None	Peprotech	500-P214	Rab IgG	Polyclonal
**IL-4R**	Biotin	BD Pharm	552508	Rat IgG2a, κ	mIL4R-M1
**Strep**	APC	BioLegend	405207		
**Xenon Rabbit IgG**	PE	Molecular Probes	Z25355		
**CD4**	BV650	BioLegend	100545	Rat IgG2a k	RM4-5
**CD45.2**	BV570	BioLegend	109833	Mouse (SJL) IgG2a, κ	104
**TER-119**	APC/Cy7	BioLegend	116223	Rat IgG2b k	TER-119
**CD49b**	APC/Cy7	BioLegend	108920	IgM k	DX5
**TCR beta**	APC/Cy7	Biolegend	109220	Armenian Hamster IgG	H57-597
**NK1.1**	Biotin	BioLegend	108704	mouse IgG2a	PK136
**Ly6G**	APC/Cy7	BioLegend	127624	Rat IgG2a k	1A8
**Ly6C**	Biotin	BioLegend	128004	Rat IgG2c, κ	HK1.4
**CD5**	Biotin	BioLegend	100604	Rat IgG2a, κ	53-7.3
**CD11c**	APCCy7	BioLegend	117324	Armenian Hamster IgG	N418
**CD3**	APCCy7	BioLegend	100222	Rat IgG2b, k	17A2
**F4/80**	Biotin	BioLegend	123106	Rat IgG2a, k	BM8
**B220**	Biotin	eBioscience	88-7774		
**ST2**	APC	eBioscience	17-9335	Rat IgG2a k	RMST2-2
**CD90.2**	PerCP	BioLegend	105322	rat IgG2b	30-H12
**IL-5**	PE	BioLegend	504304	Rat IgG1, κ	TRFK5
**GATA3**	FITC	Miltenyi	130-100-651	recom Human IgG1	REA174
**Strep**	AF780	eBiosciences	47-4317-82		

Supplementary Table 1. List of antibodies used for flow cytometry
