# Supplementary material for: IL‐33 delivery induces serous cavity macrophage proliferation independent of interleukin‐4 receptor alpha
Source: Eur J Immunol. 2016 Oct 11;46(10):2311–21. doi: 10.1002/eji.201646442 (PMC5082546; doi:10.1002/eji.201646442)
Supplement: Supplementary file 2 — Peer review correspondence [file EJI-46-2311-s002.pdf]

Peer review correspondence

European Journal of Immunology

IL-33 delivery induces serous cavity macrophage proliferation independent of interleukin-4 receptor alpha

Lucy H. Jackson-Jones, Dominik Rückerl, Freya Svedberg, Sheelagh Duncan, Rick M. Maizels, Tara E. Sutherland, Stephen J. Jenkins, Henry J. McSorley, Cécile Bénézech, Andrew S. MacDonald and Judith E. Allen

Corresponding author: Judith E. Allen, Faculty of Life Sciences, University of Manchester, Manchester, United Kingdom

Review Timeline:	Submission date:	3 April 2016
	First editorial decision:	4 May 2016
	First revision received:	30 May 2016
	Second editorial decision:	1 July 2016
	Second revision received:	5 July 2016
	Accepted:	3 August 2016

Handling Executive Committee members: Prof. Francesco Annunziato

Please note that the correspondence below does not include the standard editorial instructions regarding preparation and submission of revised manuscripts, only the scientific revisions requested and addressed.

First Editorial Decision – 4 May 2016

Dear Dr. Allen,

Manuscript ID eji.201646442 entitled "Interleukin-33 drives macrophage proliferation independently of Interleukin-4" which you submitted to the European Journal of Immunology has been reviewed. The comments of the referees are included at the bottom of this letter.

A revised version of your manuscript that takes into account the comments of the referees will be reconsidered for publication. Should you disagree with any of the referees' concerns, you should address this in your point-by-point response and provide solid scientific reasons for why you will not make the requested changes.

You should also pay close attention to the editorial comments included below. **In particular, please edit your figure legends to follow Journal standards as outlined in the editorial comments. Failure to do this will result in delays in the re-review process.**

Please note that submitting a revision of your manuscript does not guarantee eventual acceptance, and that your revision will be re-reviewed by the referees before a decision is rendered.

If the revision of the paper is expected to take more than three months, please inform the editorial office. Revisions taking longer than six months may be assessed by new referees to ensure the relevance and timeliness of the data.

Once again, thank you for submitting your manuscript to European Journal of Immunology and we look forward to receiving your revision.

Yours sincerely,
Nadja Bakocevic

On behalf of Prof. Francesco Annunziato

Dr. Nadja Bakocevic
Editorial Office
European Journal of Immunology
e-mail: ejied@wiley.com
www.eji-journal.eu

****

Reviewer: 1

Comments to the Author
Jackson-Jones and colleagues here report on the role of IL-33 in macrophage proliferation and alternative polarization in different in vivo settings.
Experimental approaches are appropriate and results are convincing and well reported. My main concern refers to the evidence of direct effects of IL-33 on macrophage proliferation. In vitro data (3F and G, probably better placed in Fig 1) overall show that the direct effect of IL-33 on macrophage proliferation is minor, only evident at high dosages, and somehow inconstant (as an example, in this set of data it is evident in F and not anymore in G). I wonder whether a survival effect should not be taken in

European Journal of Immunology

consideration, also in consideration of results shown in Fig 6. Also in consideration of this, I believe the title should be modified to avoid delivering a confusing message.

Minor requests/comments:

Figure 1: results obtained with CSF1 and IL-4, mentioned at page 6, should be shown for a matter of comparison

Figure 2: reporting panels C and D data authors claim an effect on RELM indeed not evident in these figures: the text should be modified accordingly

Fig 4 and 5 are based on a model referred to a manuscript submitted for publication. At least authors should show data indicating that Alternaria is able to induce significan IL-33 levels in the peritoneal cavity (page 10).

Reviewer: 2

Comments to the Author

In this paper Jackson-Jones et al have studied the role of IL-33 in the proliferation and alternative activation of macrophages. They assessed proliferation and activation of peritoneal and pleural macrophages by giving rIL-33 directly into the peritoneal cavity and intranasally, respectively. They also examined the direct effect of IL-33 on macrophages in vitro, and examined the effect of IL-33 on macrophages in Alternaria-induced lung inflammation and during a nematode infection using IL-33R deficient mice. Based on these experiments they conclude that IL-33 induce macrophage proliferation independent of IL-4Ra, whereas both are essential for induction of alternative activation.

Although this work is potentially interesting the in vivo experiments do not definitely prove that IL-33 act directly on macrophages. Moreover, culturing isolated macrophages with rIL-33 showed little signs of proliferation.

Major points:

1. Central to this paper is the effect of IL-33 on macrophage proliferation and the authors use expression of Ki-67 as a proliferation marker. They separate Ki-67 expression into Ki-67hi and Ki-67+ cells. The Ki-67hi cells are clearly defined in most plots. However, Ki-67+ cells appear to be arbitrary defined. For example in the same type of experiment Fig 1C shows 5.2% Ki-67+ cells whereas Fig 2A shows 48.3% + cells. To validate the results of Ki-67 the authors should show that the staining is localized to the nucleus by staining of cytospins. The flow plots should also include results after PBS as well as negative controls. The authors should also use the same type of plots in all experiments.

2. The gating strategy for RELMa also appears arbitrary with no clearly defined positive population. Negative controls should be included. The plots in Fig 3C (IL4ra-/-) are not representative for the median levels in Fig 3D.

European Journal of Immunology

3. Results from the experiments that examine the direct effect of IL-33 on macrophages are shown in Fig 3F and G. Fig 3F shows increased proliferation, but only with the highest dose. However, in a similar experiment shown in Fig 3G no effect of the highest dose was observed. Moreover, the median Ki-67 values were very different in the two experiments. This apparent difference should be discussed and the results question whether IL-33 has a direct effect macrophage proliferation.

Minor point.
The cellular source of IL-33 in the allergy and nematode models should be discussed as well as the mechanism(s) for its release.

First revision – authors' response – 30 May 2016

Reviewer: 1

Comments to the Author
Jackson-Jones and colleagues here report on the role of IL-33 in macrophage proliferation and alternative polarization in different in vivo settings. Experimental approaches are appropriate and results are convincing and well reported. My main concern refers to the evidence of direct effects of IL-33 on macrophage proliferation. In vitro data (3F and G, probably better placed in Fig 1) overall show that the direct effect of IL-33 on macrophage proliferation is minor, only evident at high dosages, and somehow inconstant (as an example, in this set of data it is evident in F and not anymore in G). I wonder whether a survival effect should not be taken in consideration, also in consideration of results shown in Fig 6. Also in consideration of this, I believe the title should be modified to avoid delivering a confusing message.

We agree that this was a weakness in the manuscript as written and have taken the reviewers comments to heart. Because data showing a direct effect of IL-33 on macrophage proliferation is lacking, we have removed Figure 3 part G and have modified the text to de-emphasise a direct effect of IL-33 on in vitro macrophage proliferation, on page 8 it now states:
«These data suggested that when provided at a high enough concentration IL-33 can either act directly on macrophages to induce their proliferation or is able to induce PEC to secrete a downstream macrophage mitogen. We failed to detect an increase in the levels of the known mitogens CSF1, CSF2 and IL-4 within the culture well (data not shown).»
We have also modified the final sentence of the discussion:
"Our new data show that IL-33 delivery in vivo can induce the proliferation of macrophages independently of IL-4Rα signaling and serve to highlight an important but often overlooked interaction between the lungs and the serous cavity in which they reside."
We have modified the title so that it now reads:

European Journal of Immunology

IL-33 delivery induces serous cavity macrophage proliferation independent of interleukin-4 receptor alpha

Minor requests/comments:

Figure 1: results obtained with CSF1 and IL-4, mentioned at page 6, should be shown for a matter of comparison.

Data showing proliferation at 48h following IL-4c injection is shown in Supplemental Figure 2. We do not have data comparing injection of CSF1 and IL-33 within the same experiment. We are concerned presenting separate CSF1 data may complicate the flow of the manuscript so we have modified the sentence on page 6 to:

"Proliferation was assessed 48 hrs later, a time point at which macrophages exhibit robust proliferation following a single delivery of either IL-4 or CSF1 (unpublished observation)."

Figure 2: reporting panels C and D data authors claim an effect on RELM indeed not evident in these figures: the text should be modified accordingly

We apologise for over-stating our data, we have modified the text on page 7 so that it now states:

'However, there was a minor trend toward a reduction in the percentage of RELMα+ macrophages in the ΔdblGATA mice (Fig 2C and D) and a significant reduction in secreted RELMα protein (Fig 2E).'

Fig 4 and 5 are based on a model referred to a manuscript submitted for publication. At least authors should show data indicating that Alternaria is able to induce significan IL-33 levels in the peritoneal cavity (page 10).

We have now uploaded a copy of the manuscript in which an increase in IL-33 is seen in the pleural lavage following Alternaria instillation (Submitted paper Figure 5A). We hope that this is the data that Reviewer 2 wanted to see, as we do not expect to see an IL-33 increase in the peritoneal cavity following intra-nasal instillation of Alternaria. This manuscript has now been provisionally accepted to Nature Communications, subject to minor revisions.

Reviewer: 2

Comments to the Author

In this paper Jackson-Jones et al have studied the role of IL-33 in the proliferation and alternative activation of macrophages. They assessed proliferation and activation of peritoneal and pleural macrophages by giving rIL-33 directly into the peritoneal cavity and intranasally, respectively. They also examined the direct effect of IL-33 on macrophages in vitro, and examined the effect of IL-33 on macrophages in Alternaria-induced lung inflammation and during a nematode infection using IL-33R deficient mice. Based on these experiments they conclude that IL-33 induce macrophage proliferation independent of IL-4Ra, whereas both are essential for induction of alternative activation.

European Journal of Immunology

Although this work is potentially interesting the in vivo experiments do not definitely prove that IL-33 act directly on macrophages. Moreover, culturing isolated macrophages with rIL-33 showed little signs of proliferation.

Major points:

1. Central to this paper is the effect of IL-33 on macrophage proliferation and the authors use expression of Ki-67 as a proliferation marker. They separate Ki-67 expression into Ki-67hi and Ki-67+ cells. The Ki-67hi cells are clearly defined in most plots. However, Ki-67+ cells appear to be arbitrary defined. For example in the same type of experiment Fig 1C shows 5.2% Ki-67+ cells whereas Fig 2A shows 48.3% + cells. To validate the results of Ki-67 the authors should show that the staining is localized to the nucleus by staining of cytospins. The flow plots should also include results after PBS as well as negative controls. The authors should also use the same type of plots in all experiments.

2. The gating strategy for RELMa also appears arbitrary with no clearly defined positive population. Negative controls should be included. The plots in Fig 3C (IL4ra-/-) are not representative for the median levels in Fig 3D.

Response to points 1 & 2:

In all experiments performed, gates for RELMα and Ki67 are based on isotype control antibodies that were included as separate fluorescence minus two control stains (with both Ki67 and RELMα antibodies being substituted for control antibodies). Plots showing the isotype control staining have now been added to Figure 1. PBS controls have been added to Figure 1 and Figure 4. We have not added representative isotype control plots to each figure in order to maintain the clarity of the figures.

With regard to the Ki-67 staining, we believe our approach of using Ki67hi is at least as informative as nuclear localization of the antibody. In addition, the image stream analysis in figure 1 A&B directly shows that macrophages are undergoing nuclear division. As the reviewer clearly is aware, Ki67 staining by itself needs to used with caution. That is the reason that we have used Ki-67hi to validate that cellular proliferation is occurring in response to specific stimuli. Our group has assessed Ki67 co-staining with BrDu following a 3hr BrDu pulse, which provides a definitive marker of proliferative events. We have shown that cells with a high level of KI67 directly overlap with BrDU staining following a short pulse (Jenkins et al. JEM 2013). This distinction is robust in multiple co-staining experiments. Phil Taylor's group has done similar experiments and shown that Ki67hi staining directly overlaps with phospho-Histone H3 (pHH3)-positive cells with 4N DNA content, an indicator of active mitosis (Davies et al EJIs 2011). Critically, not all commercially available Ki67 antibodies allow this distinction to be made. The only antibodies we have found to do so are the B56 antibody clone from BD Biosciences and the REA183 antibody clone from Miltenyi biotech. We have stated in the manuscript on page 6 “We and others have previously shown that macrophages which exhibit a high level of Ki67 expression after staining

European Journal of Immunology

with BD clone B56 are in active cell cycle [6, 17].”
With regard to RELMa, we have replaced the plots in Fig 3C to more accurately fit the median in Fig 3D. All plots have been made uniform between the figures.

3. Results from the experiments that examine the direct effect of IL-33 on macrophages are shown in Fig 3F and G. Fig 3F shows increased proliferation, but only with the highest dose. However, in a similar experiment shown in Fig 3G no effect of the highest dose was observed. Moreover, the median Ki-67 values were very different in the two experiments. This apparent difference should be discussed and the results question whether IL-33 has a direct effect macrophage proliferation.

We agree that this data is our least compelling, please see our response to reviewer 1 above.

Minor point.
The cellular source of IL-33 in the allergy and nematode models should be discussed as well as the mechanism(s) for its release.
We show in our submitted work (now uploaded as supplementary information) that the stromal cells of the fat associated lymphoid clusters (FALCs) present within the pericardium and mediastinum are a source of local IL-33 within the pleural cavity (see submitted manuscript figure 4). We predict that the release of IL-33 from these cells may be due to the increased respiratory rate that occurs during the allergy model, physically stretching the stroma. The presence of the highly motile parasite may directly damage the FALC stromal cells in the nematode model resulting in the release of IL-33. We do however have no evidence to support such hypotheses so we have not added this to the manuscript text.

Second Editorial Decision – 1 July 2016

Dear Prof. Allen,

Thank you for submitting your revised manuscript ID eji.201646442.R1 entitled "IL-33 delivery induces serous cavity macrophage proliferation independent of interleukin-4 receptor alpha" to the European Journal of Immunology. Your manuscript has been re-reviewed and the comments of the referees are included at the bottom of this letter.

Although the referees have recommended publication, some revisions to your manuscript have been requested. Therefore, I invite you to respond to the comments of the Referee 2 and revise your manuscript accordingly.

You should also pay close attention to the editorial comments included below. **In particular, please edit your figure legends to follow Journal standards as outlined in the editorial comments. Failure to do this will result in delays in the re-review process.**

If the revision of the paper is expected to take more than three months, please inform the editorial office. Revisions taking longer than six months may be assessed by new referee(s) to ensure the relevance and timeliness of the data.

Once again, thank you for submitting your manuscript to European Journal of Immunology. We look forward to receiving your revision.

Yours sincerely,
Nadja Bakocevic

on behalf of Prof. Francesco Annunziato

Dr. Nadja Bakocevic
Editorial Office
European Journal of Immunology
e-mail: ejied@wiley.com
www.eji-journal.eu

****

Reviewer: 1

Comments to the Author
No further requests.

Reviewer: 2

Comments to the Author
I thank the authors for having responded satisfactory to some of the comments, but I still do not understand why they have included the total number Ki67+ cells in many of their figures. They state the high expression of Ki67 is a good marker for proliferation, to which I agree, but they have not justified the

European Journal of Immunology

use of Ki67+ cells. There is no explanation in the Result section why the authors distinguish between Ki67+ and Ki67hi and what it means.

Second revision – authors' response – 5 July 2016

Thank you for your response. With regard to the final query by referee 2. We include Ki67+ staining for several reasons.

First Ki67 staining, although not an absolutely reliable marker of dividing cells, does give an indication of cells that are undergoing cell division, or have recently done so. We find in all our experiments that IL-33 induces a significant increase in the percent cells expressing Ki67+, so showing total Ki67 is relevant to the story.

Because, Ki67 does not uniformly represent cells that are in cell cycle we include Ki67hi+ as a confirmation that a subset of those cells are actively dividing. Because both results (total and high) are typically significant in our experiments, and because researchers are more familiar with seeing total Ki67 expression data, we feel it is valuable to show both Ki67 total and Ki67hi as the most informative and transparent means to display the data.

We already had this sentence in the results "We and others have previously shown that macrophages which exhibit a high level of Ki67 expression after staining with BD clone B56 are in active cell cycle [6, 17]."

We have now expanded this to "Ki67 staining can reflect cells that have recently proliferated rather than cells that are actively dividing. We thus additionally gated on macrophages which exhibited a high level of Ki67 expression after staining with BD clone B56. Because it has been demonstrated that these cells are in active cell cycle [6, 17], Ki67high staining provided additional confirmation that treatment induced cellular proliferation."

Third Editorial Decision – 25 July 2016

Dear Judi,

Thank you again for your prolonged patience with this particular peer review process. The referee has kept his word and submitted his report first thing this morning. I took a bit of time to finalize your text file,

which had been edited at the last decision round, and finally I can give you the positive news that was long in coming:

It is a pleasure to provisionally accept your manuscript entitled "IL-33 delivery induces serous cavity macrophage proliferation independent of interleukin-4 receptor alpha" for publication in the European Journal of Immunology. For final acceptance, please follow the instructions below and return the requested items as soon as possible as we cannot process your manuscript further until all items listed below are dealt with.

Please note that EJI articles are now published online a few days after final acceptance (see Accepted Articles: http://onlinelibrary.wiley.com/journal/10.1002/(ISSN)1521-4141/accepted). The files used for the Accepted Articles are the final files and information supplied by you in Manuscript Central. You should therefore check that all the information (including author names) is correct as changes will NOT be permitted until the proofs stage.

We look forward to hearing from you and thank you for submitting your manuscript to the European Journal of Immunology.

Yours sincerely,
Karen Chu

on behalf of Prof. Francesco Annunziato

Dr. Karen Chu
Editorial Office
European Journal of Immunology
e-mail: ejied@wiley.com
www.eji-journal.eu
